# Variants *1, *2, *3 and *17 of CYP2C19 Cytochrome among *Helicobacter Pylori* Carriers from Manaus, Amazonas State, Brazil

**DOI:** 10.31557/APJCP.2021.22.7.2289

**Published:** 2021-07

**Authors:** Everton Ricardo de Abreu Netto, Rebeca Linhares Abreu Netto, Monique Freire Santana, José Pereira de Moura Neto, Luiz Carlos de Lima Ferreira

**Affiliations:** 1 *Gastroenterology and Digestive Endoscopy Service, Manaus Adventist Hospital, Manaus, Brazil. *; 2 *School of Health Sciences, State University of Amazonas, Manaus, Brazil. *; 3 *Carlos Borborema Clinical Research Institute, Dr. Heitor Vieira Dourado Tropical Medicine Foundation, Manaus, Brazil. *; 4 *Department of Pathology, Dr. Heitor Vieira Dourado Tropical Medicine Foundation, Manaus, Brazil. *; 5 *Department of Education and Research, Amazonas Oncology Control Center Foundation, Manaus, Brazil. *; 6 *Faculty of Pharmaceutical Sciences, Federal University of Amazonas, Manaus, Brazil. *

**Keywords:** CYP2C19 polymorphism, drug metabolizers, Helicobacter pylori, Proton pump inhibitor

## Abstract

**Objective:**

The objective of the present study was to estimate the frequency of CYP2C19 cytochrome variants *1, *2, *3 and *17 among *Helicobacter pylori* carriers from Manaus, Amazonas state, who were treated at Fundação de Medicina Tropical Dr. Heitor Vieira Dourado (FMT-HVD).

**Methods:**

From the 78 recruited individuals who underwent upper gastrointestinal endoscopy with biopsy and histopathological test, 50 tested positive for *H. pylori*. Peripheral blood was collected from this group and CYP2C19 *2, *3 and *17 alleles were genotyped using qPCR.

**Results:**

Of the 50 *H. pylori* + individuals, 22 were male and 28 were female. Their age varied from 18 to 67 years old, with the mean age being 40.24. Racial groups were classified by self-declaration, based on the official Instituto Brasileiro de Geografia e Estatística (IBGE) categories: 16% (8) were white, 78% (39) were brown, 4% (2) were black, and 2% (1) were indigenous. *H. pylori* infection was classified using the system of crosses, with 30% (15) of infections being classified as mild (+), 30% (15) as moderate (++), and 40% (20) as severe (+++). The CYP2C19 genotype results showed an allelic frequency of 11% for *2 (20% of the subjects), 6% for *3 (10% of the subjects) and 17% for *17 (30% of the subjects). Based on the phenotypic profiles, the individuals were classified as poor metabolizers (PM, 10%), intermediate metabolizers (IM, 2%), extensive metabolizers (EM, 58%) and ultra-rapid metabolizers (UM, 20%).

**Conclusions:**

Even though the percentage of allele *3 was higher than expected, the percentage of allele *17, a possible contributor to *H. pylori* eradication failure, was also significant. The population that self-declared as brown showed a unique genotypic pattern, unlike any other population described in previous studies. Our results show that small populations may have genetic particularities that are relevant to therapeutic outcomes.

## Introduction

Cytochrome P450 (CYP, P450) is a hemoprotein-class cellular pigment that has been researched around the world for over 55 years, with several scientists devoting their lives to research of this pigment. The success obtained in their research has contributed to several fields of knowledge, such as the fields of medicine, agriculture and biotechnology (Yamazaki, 2014), and there have been 48,870 articles published to date (MEDSUM, on 01/20/2020).

Since the discovery of individuals with inborn errors of metabolism, it has been suspected that these individuals differ in P450 when compared to the general population, which would lead to loss of function. However, only with the advent of recombinant DNA technology has it become possible to characterize these variations (White et al., 1984). These differences are called polymorphism, which is defined as a variation of the original cytochrome, and which needs to be present in at least 1% of individuals in a population, and which is transmitted in accordance with Mendel’s law of inheritance (Kallow, 1962 in: Yamazaki, 2014).

Polymorphisms may occur through a single nucleotide variation, microdeletion or microinsertion. These variations may be neutral, cause loss or gain of functions when compared to the wild allele (Samer et al., 2013). The variants are cataloged in the Human CYP Allele Nomenclature Database, according to a standard allele nomenclature system established by the Human Genome Nomenclature Society, as the so-called “star alleles” (*) nomenclature. Each variant also receives a unique identification number (rs). This database has been categorized and reviewed by an online community of researchers and, since 2017, has been available for consultation at www.pharmvar.org, where more than 660 alleles, variants of 29 P450 superfamily genes, have been published.

Of particular interest to the present study is the enzyme CYP2C19, which so far has had 32 variants and 18 sub-variants cataloged, and variants *2 (Pro227Pro, rs4244285) and *3 (Trp212Ter, rs4986893) are of clinical interest, since they cause losses of function, and variant *17 (-806C>T, rs12248580), since it causes gains of function (Lee, 2013). CYP2C19 is responsible for the metabolization of various xenobiotics, including proton pump inhibitors (PPIs).

PPIs play an important role in the treatment for eradication of *Helicobacter pylori* (*H. pylori*), a bacterium prevalent in 44.3% of the world’s population (Zamani et al., 2018) that is recognized as the most relevant risk factor for gastric adenocarcinoma and MALT lymphoma, and the main etiological agent of chronic gastritis and peptic ulcer (Malfertheiner et al., 2017; Coelho et al., 2018). The interest in CYP2C19 is due to the fact that worldwide there has been a growing increase in therapeutic failure in eradication of *H. pylori*. This increase instigates the search for new treatments, as well as ways to improve the success of existing ones.

Since CYP2C19 variants interfere with the way it metabolizes PPIs, it is vitally important in the epidemiology of *H. pylori* infection and diagnosis to know the genotypic and phenotypic profile of our population so that we can customize the dosage of drugs currently in use. The result of this enables the optimizing of the results of the treatment instituted without requiring large investments, while on other research fronts new therapeutic schemes are sought.

Therefore, the aim of this study was to estimate the frequency of CYP2C19 cytochrome variants *1, *2, *3 and *17 among *Helicobacter pylori* carriers from Manaus, Amazonas state, Brazil who were treated at Fundação de Medicina Tropical Dr. Heitor Vieira Dourado (FMT-HVD), in order to provide information regarding the genotypic and phenoctypic profiles of this population.

## Materials and Methods

An observational cross-sectional study was performed. Participants were selected without distinction of gender, color, health status, class or social group, respecting the inclusion and non-inclusion criteria, from patients referred to the Digestive Endoscopy Service at FMT-HVD for upper GI endoscopy due to suspected *H. pylori* infection.

Between August 2018 and May 2019, 78 individuals underwent upper GI endoscopy with a biopsy and subsequent histopathological testing. From these, 50 individuals who tested positive for *H. pylori* infection were selected. In this group, molecular technics were performed to search for CYP2C19 *2, *3 and *17 genotypes using qPCR.

Adults of either genders, aged between 18 and 70 years old, referred for upper endoscopy with gastric biopsy were submitted to a preliminary interview, during which they were invited to participate in the study. After being properly instructed, the individuals who agreed to participate in the study signed the ICF and had their procedure scheduled.

Patients submitted to previous treatment for *H. pylori*; with previous gastric surgery; immunosuppressed; who had used antimicrobials within 30 days prior to performing the upper endoscopy; currently using anticoagulants (to prevent gastric biopsies), or non-*H. pylori* carriers, with an absence of infection confirmed by histopathological examination of antral and gastric body mucosa fragments were not included.

During the endoscopic procedure, four biopsy fragments (1-3 mm) were collected; one at the greater curvature and one at the lesser curvature of the gastric body, for histopathological analysis for the presence of *H. pylori*. The specimens for histopathological analysis were placed in 10% buffer formalin solution and routinely processed. The hematoxylin and eosin stain and Warthin-Starry stain were used for identification of *H. pylori*. The quantification of chronic inflammation and activity of gastritis and of *H. pylori* was performed according to the updated Sydney classification (Dixon et al., 1996), i.e., mild (1+), moderate (2+) and severe (3+).

Upon receipt of the biopsy report and therapy, the *H. pylori* carriers, who had the presence of infection confirmed by histopathological examination of antral and gastric body mucosa fragments, had peripheral blood collected and stored for later analysis of the CYP2C19.

Approximately 3.0 mL of peripheral blood were collected in a tube with disodium EDTA as the anticoagulant and, from it, an aliquot (~200 µL) was kept in 1.5 mL tubes for extraction of nuclear DNA. The DNA was extracted using a QIAamp DNA Mini Kit (Qiagen, Hilden, Germany) according to the manufacturer’s protocol (Cat No./ID 51304). After extraction, the DNA was quantified with a spectrophotometer (NanoDrop™ 2000, Thermo Fisher Scientific, USA), and then stored at -20 ºC. 

SNP genotyping was conducted using TaqMan® SNP genotyping assays (Applied Biosystems, USA) on a PCR System (QuantStudio™ 6 Flex Real-Time, Applied Biosystems, USA). The probes used were CYP2C19*2 (Pro227Pro) rs4244285; CYP2C19*3 (Trp212Ter) rs4986893; and CYP2C19*17 (-806C>T) rs12248560. The genotypic frequencies for the polymorphisms were analyzed according to what would be expected by Hardy-Weinberg equilibrium.

Samples for which none of the three genotyped alleles (*2, *3 and *17) were found were considered type *1 (wild), since the other alleles described in the literature have a statistically non-significant occurrence among the general population (Samer et al., 2013; Zhou et al., 2017).

Statistical analysis was performed using Stata SETM 13 software (Stata Corp, USA). P-values of <0.05 were considered significant. The analysis of the qualitative or categorical variables of three or more groups was performed using the non-parametric Chi-square test (x^2^), and corrected by the Mantel-Haenszel and Yates tests.

## Results

Of the 50 volunteers selected, 22 were male (44%) and 28 were female (56%). The age of the volunteers ranged between 18 and 67, with mean age of 41.55±15.60 years old for males and 39.21±13.37 for females.

In regards to ethnicity, the participants were classified according to self-declarations, based on the official IBGE categories. As such, 8 (16%) declared themselves as white, 39 (78%) as brown, 2 (4%) as black and 1 (2%) as indigenous and none declared themselves as yellow.

The main complaints that led patients to seek medical attention were epigastric pain (in 29/58% of cases), dyspepsia (in 10/20% of cases) and heartburn in 5/10% of cases. This distribution, which is stratified by gender, can be seen in Table 1. When the main complaints were analyzed by gender, it was observed that 80% of dyspepsia complaints occurred among males (p=0.007), while 65.5% of epigastric pain complaints occurred among females, but not significantly (p=0.152).

When correlating the patients’ complaints with the bacillary density of *H. pylori* found in the histopathological studies, it was observed that the reported complaints had no influence in the distribution of cases (Supplementary Table 1). The most frequent endoscopic findings were mild enanthematous pangastritis (24%), followed by moderate enanthematous exudative pangastritis (20%), and mild antral enanthematous gastritis (16%), while 40% had other causes.

The histopathological findings were severe chronic superficial gastritis (54%), moderate chronic superficial gastritis (42%) and mild chronic superficial gastritis (4%). The bacillary density of *H. pylori* in the histopathological studies indicated 20 severe cases (40%), 15 moderate cases (30%) and 15 mild cases (30%). Table 2 shows the different endoscopic and histopathological diagnoses found in the volunteers.

When analyzing the distribution of the histopathological findings according to the bacillary density of *H. pylori*, a significant association among the severity of the inflammation of the mucosa and the infection was found (p=0.009) (Supplementary Table 2).

The genotyping results can be seen in Tables 3 to 5. Table 3 describes the results of genotyping for CYP2C19 variants *2, *3 and *17 among the patients. The correlation of these genotypes with the patient’s ethnicity, the intensity of the gastritis as described by histopathological studies and the bacillary density of *H. pylori* can be seen in Table 4.

Genotyping results described for each individual patient and correlated with their ethnicity can be seen in Supplementary Table 3. When classifying the genotypic findings according to the possible phenotypic expressions, we found 5 PMs (10%), 1 IM (2%), 29 EMs (58%) and 10 UMs (20%), as shown in Table 5.

**Table 1:** Complaints Reported by Patients According to Gender

Complaint Reported	Male n (%)	Female n (%)
Epigastric pain	10 (34.5)	19 (65.5)
Dyspepsia	8 (80.0)	2 (20.0)
Heartburn	2 (40.0)	3 (60.0)
Dysphagia	-	1 (100.0)
Fullness	-	1 (100.0)
Globus	-	1 (100.0)
Reflux	1 (100.0)	-
Relative with gastric cancer	-	1 (100.0)
Vomiting	1 (100.0)	-
Total	22 (44.0)	28 (56.0)

**Table 2:** The Different Endoscopic and Histopathological Diagnoses

Endoscopic diagnostics	Superficial Chronic Gastritis		
	Severe	Moderate	Mild
1	08 (67%)	03 (25%)	01 (8%)
2	05 (50%)	05 (50%)	-
3	04 (50%)	04 (50%)	-
4	03 (50%)	02 (33%)	01 (17%)
5	02 (50%)	02 (50%)	-
6	02 (67%)	01 (33%)	-
7	01 (100%)	-	-
8	-	01 (100%)	-
9	-	01 (100%)	-
10	-	01 (100%)	-
11	01 (100%)	-	-
12	01 (100%)	-	-
13	-	01 (100%)	-
Total	27 (54%)	21 (42%)	02 (4%)

Endoscopic diagnostics; 1, Mild enanthematous pangastritis; 2, Moderate enanthematous exudative pangastritis; 3, Mild enanthematous gastritis of antrum; 4, Moderate enanthematous pangastritis; 5, Mild enanthematous exudative pangastritis; 6, None; 7, Mild exudative gastritis of antrum; 8, Mild enanthematous gastritis of corpus; 9, Mild raised erosive gastritis of antrum; 10, Mild flat erosive gastritis of antrum; 11, Severe nodular gastritis of antrum; 12, Moderate nodular gastritis of antrum; 13, Severe raised erosive pangastritis

**Table 3:** Frequency of CYP2C19 Analyzed Variants

Genotypes	CYP2C19 n (%)		
	*2	*3	*17
Homozygote	01 (2.0%)	01 (2.0%)	02 (4.0%)
Heterozygote	09 (18.0%)	04 (8.0%)	13 (26.0%)
Wild Type	40 (80.0%)	45 (90.0%)	35 (70.0%)

n, cases

**Table 4:** Allele Frequencies According to Ethnicity, Intensity of Gastritis and Quantity of *H. pylori*

		Allele Frequency								
		*2 N (%)			*3 n (%)			*17 n (%)		
		HO	HT	WT	HO	HT	WT	HO	HT	WT
Ethnicity	Wh	-	1 (12.5)	7 (87.5)	-	-	8 (100.0)	-	4 (50.0)	4 (50.0)
	Br	1 (2.6)	7 (17.9)	31 (79.5)	1 (2.6)	4 (10.3)	34 (87.2)	2 (5.1)	7 (17.9)	30 (76.9)
	Bl	-	1 (50.0)	1 (50.0)	-	-	2 (100.0)	-	1 (50.0)	1 (50.0)
	Ind	-	-	1 (100.0)	-	-	1 (100.0)	-	1 (100.0)	-
Intensity of gastritis	+++	-	8 (16.0)	19 (38.0)	-	4 (8.0)	23 (46.0)	2 (4.0)	7 (14.0)	18 (36.0)
	++	1 (2.0)	1 (2.0)	19 (38.0)	1 (2.0)	-	20 (40.0)	-	5 (10.0)	16 (32.0)
	+	-	-	2 (100.0)	-	-	2 (4.0)	-	1 (2.0)	1 (2.0)
Bacillary density	+++	-	3 (20.0)	12 (80.0)	-	1 (6.7)	14 (93.3)	1 (6.6)	4 (26.7)	10 (66.7)
	++	1 (6.7)	2 (13.3)	12 (80.0)	1 (6.7)	2 (13.3)	12 (80.0)	-	4 (26.7)	11 (73.3)
	+	-	4 (20.0)	16 (80.0)	-	1 (5.0)	19 (95.0)	1 (4.0)	5 (26.0)	14 (70.0)

n, cases; WT, Wild Type; HT, heterozygote; HO, homozygote; Wh, white; Br, brown; Bl, black; Ind, indigenous; severe, + + +; moderate, + + ; mild, +

**Table 5:** CYP2C19 Genotypic Findings and the Predicted Phenotypes

2	Genotypes		
PM	*2/*2	*2/*3	*3/*3
	01 (2%)	03 (6%)	01 (2%)
IM	*1/*2	*1/*3	
	01 (2%)	-	
EM	*1/*1		
	29 (58%)		
UM	*17/*17	*1/*17	
	02 (4%)	08 (16%)	
Uncertain	*2/*17	*3/*17	*2/*3/*17
	04 (8%)	-	01 (2%)

PM, poor metabolizer; IM, intermediate metabolizer,; EM, extensive metabolizer; UM, ultra-rapid metabolizer

## Discussion

This monocentric study analyzed a convenience sample of 50 patients with dyspeptic symptoms treated in Manaus who were found to be infected by *H. pylori*. The aim was to determine the frequency of CYP2C19 *1, *2, *3 and *17 among them.

Boyanova and Mitov (2010) found a prevalence of 20-50% failure in the different first-line treatment regimens for *H. pylori*. One of the causes of this high rate of failure is the CYP2C19 polymorphism (Kuo et al., 2012). Knowing the metabolic profile of the patients infected with *H. pylori* can be an important tool in choosing the most appropriate therapeutic regimen, thus increasing the chance of its successful eradication.

While alleles *2 and *3 cause loss of function, with slower metabolism of PPIs compared to the wild allele (*1), allele *17 causes a gain in function, accelerating PPI metabolization and decreasing their effectiveness in maintaining an optimal intragastric pH for antibiotic action. Therefore, poor and intermediate metabolizers (when compared to extensive metabolizers) are expected to have equal or greater rates of treatment success, while ultra-rapid metabolizers may have a higher failure rate.

In his review article, Lee (2013) found 10 studies performed among whites, with the presence of the allele *2 ranging from 11.4-20.8% of the subjects studied, the allele *3 between 0.0-0.8% (not determined in 5 studies), and the allele *17 between 15.4-27.2% (not determined in 2 studies). He found 6 studies performed among blacks, with the allele *2 present in 11.0-25.0% (determined in 4 studies), allele *3 present in 0.0-0.8% (in the same 4 studies), and the allele *17 in 17.9-21.0% of the subjects tested (determined in 2 studies). He also found 14 studies conducted among Asians, where 12.0-37.5% of individuals presented allele *2 (13 studies), 0.0-12.8% presented allele *3 (same 13 studies), and allele *17 was found in 0.5 -4.4% of the subjects (6 studies). No brown population is described.

In our study group, we found a frequency of 11% of allele *2 distributed in 10 individuals, 6% of allele *3 in 5 individuals and 17% of allele *17 in 15 individuals. In this group, 16% declared themselves as white, 78% as brown, 4% as black, 2% as indigenous, and none declared themselves as Asian.

When comparing these data with Lee’s findings, we observe that the highest percentage of the allele *2 was 37.5% among Asians, 25% among blacks and 20.8% among whites, placing our group’s profile within all groups studied by Lee.

Allele *17 was the least researched, with up to 4.4% of presence among Asians, 21% of blacks, and 27.2% of whites studied. Comparing these findings to our 17% finding, our group has a profile closer to the black population.

Allele *3 was present in 12.8% among Asians, but in only 0.8% of black and white individuals, which places the results of this study between both groups. When comparing this finding with that of other Brazilian studies, we see that Santos et al., (2011) studied 1,212 individuals divided into indigenous, white, brown and black, and found no carrier of the allele *3. Suarez-Kurtz et al., (2012) studied 1,034 subjects divided into whites, browns and blacks, and found only 1 individual with the allele *3 (0.05%). Kohlrausch (2014) researched 179 individuals from a southern Brazilian population for alleles *2 and *3, and did not find allele *3 either.

However, Vargens et al., (2012) reported the finding of 11.5% of allele *3 carriers in a group of 50 Brazilians with >50% indigenous ancestry, a number closer to Lee’s findings among some Asian populations, such as the Japanese and Korean. Although 78% of the individuals in the current study declared themselves as brown and only 2% as indigenous, it was possible to observe the characteristic features of indigenous people in many of these individuals, which historically are descendants of the miscegenation that occurred between natives and immigrants. This leads us to believe that many of them may have indigenous ancestors in their families. It is our hypothesis that there may be a relationship between the frequency of the allele *3 found in this study and the probable indigenous ancestry of these individuals.

Even though the percentage of the allele *3 found was higher than expected, we found a significant percentage of the allele *17, which is responsible for the fast metabolization of the PPIs, and possibly contributes to a higher failure rate in the eradication of *H. pylori*.

When correlating the intensity of gastritis and the density of *H. pylori* found in the histopathological exams, we see a directly proportional association (p=0.009), i.e., the greater the amount of *H. pylori* present, the greater the inflammation. On the other hand, when correlating the different alleles of the individuals and the *H. pylori* density found or the intensity of gastritis, no significant association was found.

The present study shows us that small populations may have genetic particularities when compared to larger populations. This confirms the findings of a systematic review recently published by Petrović (2020), which includes nationwide studies from only 20 to more than 40,000 individuals. This review shows that there is no homogeneous distribution of alleles among the populations of different European countries, and these variations may influence the results of the therapies.

Therefore, special attention must be paid to the brown population from the Amazonas state, where we observed no tendency of a genotypic pattern closer to either Asian, black or white populations as described in previous studies (Lee, 2013). This study demonstrates that the local brown population, as a result of its miscegenation with the native Amazonians with white, black and Asian immigrants, has a unique pattern.

The small sample size of this study was the main limitation, but, despite this, through our findings we were able to detect the influential polymorphic variants for the first time in an Amazonian population, their relationship with *H. pylori* density and the severity of the histologic properties of the gastritis.

The results obtained cannot be extrapolated to the general population, but may be used as a reference in decision making. Further studies with larger sample sizes are required to understand the influence of these polymorphic variants in the response to pharmacogenetic treatment.

In conclusion, it was found that certain populations have specific genotypic characteristics that can influence the treatment for eradication of *H. pylori*, a key point in the prevention of relevant gastric pathologies. Furthermore, it is necessary to perform further studies to elucidate the pattern of the studied polymorphisms in other populations. The knowledge of the impact of CYP variants in the metabolization and consequent drug efficiency is of utmost importance for clinical practice.

## Author Contribution Statement

Conceived and designed the experiments: ERAN, MFS, JPMN, LCLF. Acquisition of data: ERAN, RLAN, MFS, JPMN, LCLF. Data analysis: ERAN, RLAN, MFS, JPMN, LCLF. Wrote the paper: ERAN, RLAN, JPMN. Critical revision of the manuscript: ERAN, RLAN, MFS, JPMN, LCLF. All authors read and approved the final manuscript.
